# Comparison of predicted aquatic risks of pesticides used under different rice-farming strategies in the Mekong Delta, Vietnam

**DOI:** 10.1007/s11356-016-7991-4

**Published:** 2016-11-16

**Authors:** Nadja Stadlinger, Håkan Berg, Paul J. Van den Brink, Nguyen. T. Tam, Jonas S. Gunnarsson

**Affiliations:** 1 Department of Ecology, Environment and Plant Sciences, Stockholm University, Stockholm, Sweden; 2 Department of Physical Geography, Stockholm University, Stockholm, Sweden; 3 Department of Aquatic Ecology and Water Quality Management, Wageningen University, Wageningen, the Netherlands; 4 Alterra, Wageningen University and Research Centre, Wageningen, the Netherlands; 5 Department of Aquaculture, Nong Lam University, HCM City, Vietnam

**Keywords:** Risk assessment, Plant protection products, Rice, Pesticide management, Fish, Integrated pest management, PRIMET, Species sensitivity distribution

## Abstract

This study evaluates the risks of pesticides applied in rice-fish and rice farming, with and without integrated pest management (IPM) strategies, to non-target aquatic organisms in two provinces of the Mekong Delta, Vietnam. Pesticide inventories and application patterns were collected from 120 Vietnamese farmers through interviews. Risks were assessed using (1) Pesticide RIsks in the Tropics to Man, Environment, and Trade (PRIMET), a first-tier model, which calculates predicted environmental concentrations (PECs) of pesticides in the rice field, based on the compound’s physico-chemical properties and the application pattern, and then compares the PECs to safe concentrations based on literature data, and (2) species sensitivity distribution (SSD), a second-tier assessment model using species sensitivity distributions to calculate potentially affected fraction (PAF) of species based on the PECs from PRIMET. Our results show that several of the used insecticides pose a high risk to fish and arthropods and that the risks are higher among rice farmers than among rice-fish farmers. This study indicates that the PRIMET model in combination with SSDs offer suitable approaches to help farmers and plant protection staff to identify pesticides that may cause high risk to the environment and therefore should be substituted with safer alternatives.

## Introduction

**Fig. 1:**
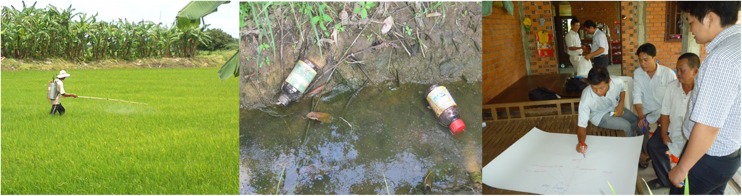
Examples of pesticide application, disposal, and IPM training in the Mekong Delta. Farmer spraying his rice field (*left*), disposed pesticide containers in a ditch (*middle*), and farmers involved in IPM training (*right*)

Vietnam is currently ranked as the fifth largest rice producer in the world, with most of its rice being farmed in the Mekong River Delta (Ricepedia 2016). The use of pesticides has helped to increase rice yields but has also led to an increased pollution that presents a potential toxicity threat to the environment and public health (Carvalho et al. 2008; Berg and Tam 2012; Tam et al. 2015a, b). A recent study found high concentrations of pesticides in sediment and drinking water of the Mekong Delta, indicating that pesticides may pose a chronic exposure risk to biota and humans (Toan et al. 2013). Several programs have been launched since 1992 in Vietnam in order to train rice farmers to reduce their pesticide use, such as integrated pest management (IPM) programs (Fig. 1). In 2005, about one million farmers had received training through Farmer Field Schools corresponding to more than 10% of all rice-farming households in the country (Van de Fliert et al. 2007). Several information campaigns on safer pesticide use have also been launched in order to help famers reduce their pesticide use in the delta (Huan et al. 1999, 2008). Some farmers practice integrated rice-fish culture in the delta that can provide a sustainable alternative to rice monocultures, as these farmers tend to decrease their use of pesticides for an optimized production of both rice and fish (Berg 2002; Berg and Tam 2012). This can also help to spread the farmers’ economic risks since the fish provides an extra important source of income. It is considered as a low external input system (Lu and Li 2006) that enhances ecosystem services for the benefit of local people’s livelihood and well-being (Berg et al. 2012). Integrated rice-fish farming is, however, vulnerable to the negative side effects of pesticides as fish are stocked directly in the rice fields. Earlier studies indicate that a high use of pesticides may impact fish growth and survival (Berg 2002; Tam et al. 2015a; Tam et al. 2016b). This could be through direct toxic effects on the fish but also through indirect impacts on different ecosystem services provided by the rice-field environment that helps to sustain a high production of both rice and fish. IPM is therefore seen as an important complementary strategy to make rice-fish farming an economic competitive alternative to rice monocultures (Berg 2002).

**Fig. 2:**
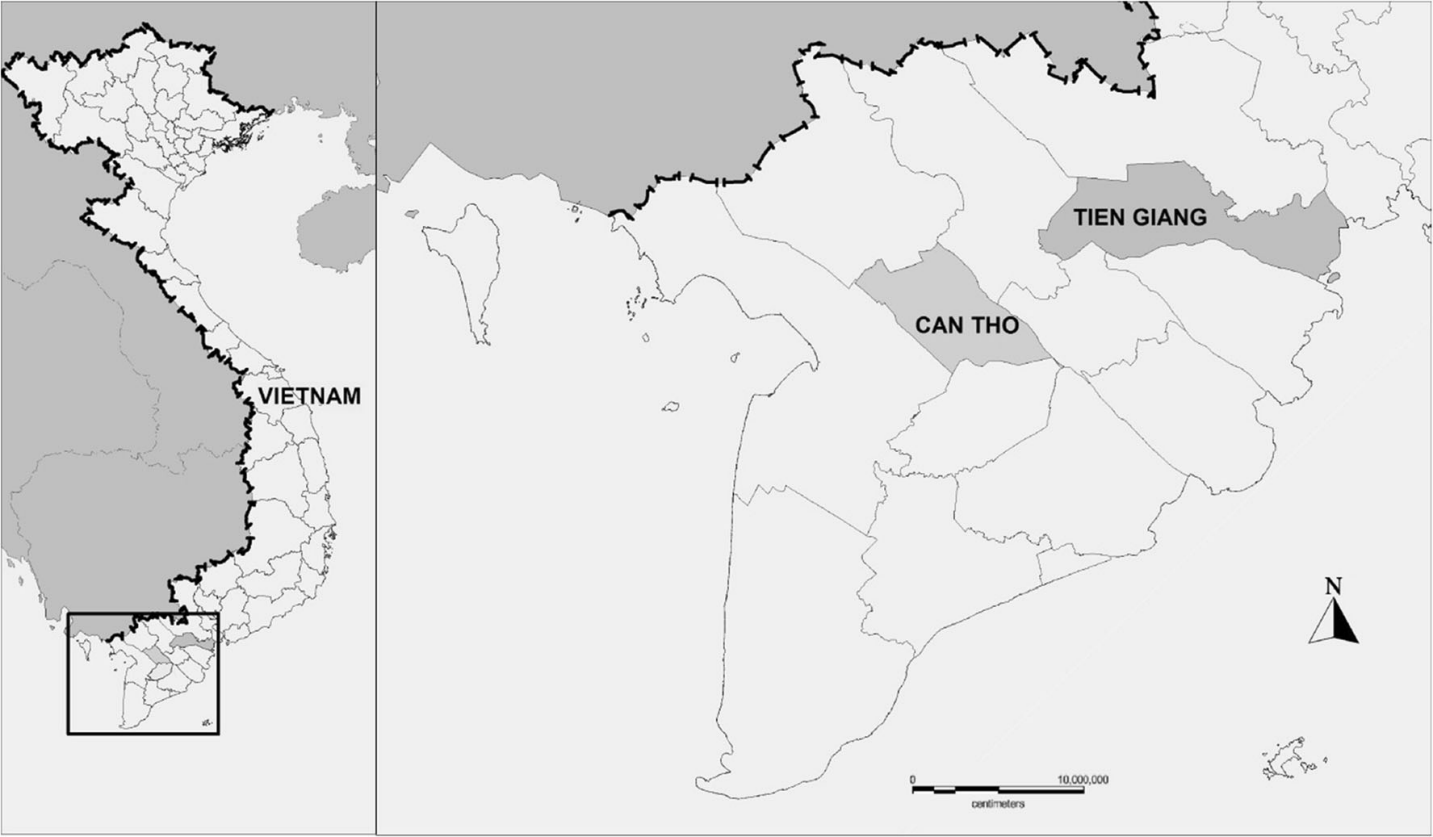
Map showing the location of the provinces Can Tho and Tien Giang in the Mekong Delta of Vietnam

The Vietnamese Mekong Delta covers an area of 40,000 km^2^ and is the most important agricultural region of Vietnam (Fig. 2). Covering only 12% of Vietnam’s total land area, it supplies more than half of the national rice output and provides 90% of Vietnam’s rice exports (General Statistics Office 2009; Johnston et al. 2010). Rice yields are the highest of the country in this region, with up to 15 metric tons per hectare and year (Cao 2011). The average farm size is between 1.0 and 1.8 ha (Sanh et al. 1998; Bosma et al. 2009; Berg and Tam 2012). Chemical fertilizers are more commonly applied by the farmers than organic fertilizers. Pesticides are commonly used with an average dose between 1 and 1.5 kg of active ingredient per hectare and crop (Duong et al. 2005; Berg and Tam 2012).

Large areas of the Mekong Delta are suitable for freshwater aquaculture, but historically, only minor areas have been used for this purpose (Nhan et al. 2007). Recently, however, aquaculture has been expanding rapidly (Nhan et al. 2007; General Statistics Office 2007; Phan et al. 2009), especially catfish farming (De Silva and Phuong 2011). The delta accounts for 70% of Vietnam’s aquaculture production (Johnston et al. 2010), which calls for a more restrictive use of pesticides and other agro-chemicals to assure an acceptable water quality for a sustainable and healthy fish production (cf. Nhan et al. 2007; Andrieu et al. 2015). The climate in the delta is characterized as tropical semi-equatorial with an annual average temperature of 27 °C. The mean annual rainfall is 1600 mm, and approximately 90% of the rain come during the rainy season in May to October (General Statistics Office 2009).

This study aims to evaluate if the use of pesticides among rice farmers and rice-fish farmers in the Mekong Delta poses a risk for direct toxicological effects on aquatic organisms living in the rice fields, with a focus on fish. The study also aims to evaluate if this risk differs between different farming strategies such as rice monoculturing, IPM, and integrated rice-fish farming. The risk is estimated using the screening model tool Pesticide RIsks in the Tropics to Man, Environment and Trade (PRIMET) (Peeters et al. 2008), which is a decision support system for assessing pesticide risks in the tropics. PRIMET is a freely available program, which needs a minimum of pesticide input data. It is designed to be used in tropical countries, taking into account temperature-dependent pesticide parameters. PRIMET can evaluate pesticide risks to aquatic life, earthworms, bees, non-target arthropods, drinking water, and dietary exposure (Peeters et al. 2008). To our knowledge, PRIMET has never been used to assess the risks of pesticide use under different rice-farming scenarios in the tropics before. Risks were further investigated using the species sensitivity distribution (SSD) model (Posthuma et al. 2002), in order to more specifically assess the risks for fish. The current trend in the delta is towards an intensified production of both fish and rice, and this is a first attempt to estimate if the current use of pesticides may pose risks to fish and other non-target aquatic organisms and, thus, be a problem for a sustainable production of both rice and fish in the Mekong River Delta.

## Material and methods

Pesticide use among rice farmers in the Can Tho City and Tien Giang Provinces of the Mekong Delta in southern Vietnam was assessed through questionnaires and interviews (Berg and Tam 2012). The study involved 120 farmers divided into conventional rice farmers (R), conventional rice-fish farmers (RF), rice farmers with training in integrated pest management (RIPM), and rice-fish farmers with training in integrated pest management (RFIPM). Each interview group in each province consisted of approximately 15 farmers. IPM farmers were defined as farmers, who had attended some form of training in IPM. Interviews were made with farmers, who had two or three rice crops per year, as these are the systems with the highest use of pesticides (Berg and Tam 2012). In this paper, we conducted a risk assessment of the pesticides identified in Berg and Tam (2012) to non-target aquatic organisms, i.e., fish and aquatic arthropods living in the rice-fish-farming system and compare the predicted risks among the four farming systems (R, RF, RIPM, RFIPM).

### Study area

The Tien Giang Province and Can Tho City (same administrative level as a province) were selected as study sites because they are two typical areas for rice and rice-fish farming in the Mekong Delta (Berg 2002; Nga and Sinh 2008). The districts of Go Cong Tay (272 km^2^) and Cai Be (421 km^2^) represent two different rice-producing areas within the Tien Giang Province. The dominant farming systems in the Cai Be District are rice and fruit cultivation, while rice and vegetable cultivation are dominant systems in the Go Cong Tay District. The areas around Cai Be are well irrigated through a network of many canals and natural rivers. The first rice crop is usually grown from November to February (the winter-spring crop), the second from February to May (the summer-autumn), and the third from May to August (the autumn-winter crop). Go Cong Tay has a much poorer irrigation system compared to the Cai Be Province. The first rice crop is grown from November to January, the second from May to August, and the third from September to November. The O Mon (126 km^2^) and Co Do (403 km^2^) Districts, which lie in Can Tho City, are also representative of the irrigated rice areas of the Mekong Delta, both in aspects of physical environment and productivity. Rice is cultivated in the dry and wet seasons using the double rice-cropping system and in the dry, spring-summer, and wet seasons using a triple rice system. Fish is usually farmed either together with the rice or in the flooded season, when the rice has been harvested. There is usually only one fish harvest per year. The most commonly farmed species are silver carp, common carp, silver barb, climbing perch, snakeskin gourami, and *Nile tilapia* (Berg 2002; Cao 2011).

### Pesticide application input data

The interview data from Berg and Tam (2012) was compiled into recorded active ingredients and amounts of applied active ingredients per hectare and crop. Pesticide products with more than one active ingredient were separated into active ingredients and percentage based on producers’ information. All application data was divided into single-application scenarios of an active ingredient per rice field and season (up to three crops per year). If one active ingredient was repeatedly used on one rice field and crop, it was included as a repeated application (*n* > 1), and the time interval between applications (Δ*t*) was included (Table 1). In case the time interval between applications was missing in the interview data, a calculated average of days between applications for insecticides, herbicides, and fungicides, respectively, was used. The data was coded so that farmer ID, management regime, rice field, and crop were traceable for each application scenario. The input data sheet resulted in approximately 700 individual application scenarios (Table 3).

**Table 1:** Pesticide application input parameters in PRIMET, where *M*, *n*, and Δ*t* vary with each application scenario

Parameter	In PRIMET	Source
Individual dose applied (g A.I./ha)	*M*	Interview data
Percentage spray drift (%)	Drift	Worst-case estimation
Percentage spray drift to ditch (%)	Drift-ditch	Worst-case estimation
Number of applications (−)	*n*	Interview data
Time interval between applications (day)	Δ*t*	Interview data

### PRIMET

The data on pesticides’ use among farmers was used as input data for the PRIMET model, which is “a decision support system for assessing Pesticide RIsks in the Tropics to Man, Environment, and Trade” (Peeters et al. 2008). The model calculates an exposure toxicity ratio (ETR = predicted environmental concentration (PEC)/predicted no effect concentration (PNEC)) for different scenarios of pesticide applications on a model water body with physical parameters that can be predefined. ETR values are classified as <1 = “no risk,” 1–100 = “possible risk,” and >100 = “definite risk” based on worst-case assumptions. Both acute and chronic risk quotients are obtained from PRIMET. The acute ETR value (ETR*n*_water-acute_) is obtained from concentrations based on *n* applications (PEC*n*_water_) and acute EC_50_ values for fish, crustaceans (*Daphnia*), and algae (PNEC _water-acute_). The chronic ETR value (ETR _water-chronic_) is derived from the lowest coefficient of either the average water concentration for fish (28 days) or *Daphnia* (21 days), divided by the respective chronic no effect concentration (Peeters et al. 2008). The physical input parameters were determined based on in situ measurements and literature estimates from the Mekong Delta area (Table 2). The pesticide application doses were derived from the interviews with farmers from the four pesticide management types (R, RF, RIPM, RFIPM) (Table 3). Spray drift to ditch (Table 2) was set to 100%, as the PRIMET model does not include runoff and thereby underestimates the amount of pesticide that reaches the water (Peeters et al. 2008). Also, rice fields are wetlands in direct contact with water, which increases the exposure of aquatic organism to pesticides sprayed on rice compared to other crops such as vegetables and fruits. The PRIMET active ingredient (A.I.) database was completed and updated with information on pesticide characteristics from the footprint Pesticide Properties Database (Lewis et al. 2016). If the half-life for water/sediment was missing, a conservative estimate of 999 days was used. This parameter is needed for calculations of the risks to the aquatic life in PRIMET but is not always available for all substances. All scenarios were analyzed in PRIMET version 3.0. The calculated PECs from PRIMET were then also used to estimate the potentially affected fraction (PAF) using the SSDs for the pesticides of the highest concern.

**Table 2:** Physical scenario parameters of the water body/rice field as defined in PRIMET

Parameter	Value	Source
Bottom width of the water body (m)	100	Field estimation
Depth of water body (m)	0.34	Field estimation
Length of water body (m)	100	Field estimation
Mass fraction organic matter in suspended solids (g/g)	0.25^a^	Average organic content (%) (low-exchange pond; Hoa et al. 2003)
Mass concentration of suspended solids in water (kg/L)	0.0001^a^	100 mg/L (Sebesvari et al. 2012)
Ambient temperature in the scenario (K)	301^a^	Average temperature (low-exchange pond; Hoa et al. 2003)
Side slope	0.33	Field estimation
Flow velocity (m/day)	0.001	Field estimation

^a^Data taken from literature source

**Table 3:** All active ingredients identified from the interview study, their occurrence in each province and per management regime, number of users and single-application scenarios, and how they were further assessed

Active ingredient	Use type^a^	Chemical class^b^	Occurrence (province)^c^	Occurrence management regimes^d^	No. of users	No. of application scenarios^e^	Assessed with PRIMET	Assessed with SSD
Alpha-cypermethrin	I	Pyrethroid	CT	R, RIPM, RF, RFIPM	8	22	x	x
Bensulfuron-methyl	H	Sulfonylurea	TG	RF	1	6	–	–
Buprofezin	I	Unclassified	CT, TG	R, RIPM, RF, RFIPM	11	21	x	–
Butachlor	H	Chloroacetanilide	TG	R, RIPM, RF, RFIPM	17	50	x	–
Cartap	I	Nereistoxin	CT, TG	R, RF, RFIPM	10	30	–	–
Diazinon	I	Organophosphorus	CT	R, RF	2	4	x	x
Ethoxysulfuron	H	Sulfonylurea	TG	R, RIPM	2	6	x	–
Etofenprox	I	Pyrethroid ether	CT	R, RIPM, RFIPM	4	10	x	x
Fenclorim^f^	H	Unclassified	CT, TG	R, RIPM, RF, RFIPM	21	49	x	–
Fenobucarb	I	N-methyl carbamate	CT, TG	R, RIPM, RF, RFIPM	55	154	x	x
Fenoxaprop-P-ethyl	H	Aryloxyphenoxy propionic acid	CT, TG	R, RIPM, RF	3	8	x	–
Fipronil	I	Pyrazole	CT	R, RIPM, RF, RFIPM	7	17	x	x
Glyphosate	H	Phosphonoglycine	CT	RIPM	1	1	x	–
Hexaconazole	F	Azole	CT, TG	R, RIPM, RF, RFIPM	43	113	x	–
Imidacloprid	I	Neonicotinoid	CT, TG	R, RIPM	3	12	x	–
Iprodione	F	Dicarboximide	TG	R, RF	2	6	x	–
Isoprocarb	I	N-methyl carbamate	CT	R, RIPM, RF	3	6	–	–
Isoprothiolane	F	Unclassified	CT, TG	R, RIPM, RFIPM	5	19	x	–
Permethrin	I	Pyrethroid	CT	R	1	4	x	x
Phenthoate	I	Organophosphorus	TG	R	1	3	x	x
Pretilachlor	H	Chloroacetanilide	CT, TG	R, RIPM, RF, RFIPM	21	49	–	–
Propiconazole	F	Azole	CT, TG	R, RIPM, RF, RFIPM	20	53	x	–
Pyrazosulfuron ethyl	H	Sulfonylurea	TG	R, RF	2	6	x	–
Quinalphos	I	Organophosphorus	CT	R, RF	3	6	x	x
Quinclorac	H	Unclassified	TG	R, RF	2	5	x	–
Thiamethoxam	I	Neonicotinoid	CT	R, RIPM, RF, RFIPM	16	54	x	–
Tricyclazole	F	Azole	TG	RFIPM	2	6	x	–
Validamycin	F	Antibiotic	CT, TG	R, RIPM, RF	6	14	–	–

^a^ *I* insecticide, *H* herbicide, *F* fungicide

^b^From PAN Pesticide Database (2014)

^c^ *CT* Can Tho, *TG* Tien Giang

^d^ *R* rice farming, *RIPM* rice farming with integrated pest management training, *RF* rice-fish farming, *RFIPM* rice-fish farming with IPM training

^e^Single pesticide application, input data in PRIMET

^f^Fenclorim (herbicide safener) is a mixture with pretilachlor (Sofit)

### SSDs

To further evaluate the risks for aquatic organisms within the rice fields, SSD curves were calculated for substances with estimated ETR acute values above 100 (Tables 3 and 4). Toxicity data for each substance was primarily collected from US EPA Ecotox Database (2016) and De Zwart (2002). In some cases, the PAN Pesticide Database (2014) was used to complement the other two databases. Identical records from multiple databases were removed, and no values given as ranges or “higher/lower than” were used. For two insecticides with plenty of fish toxicity data available, the data set was narrowed down to only include fish families of fish cultivated in the Mekong Delta. Only freshwater lab tests with LC_50_ values or EC_50_ values for immobility were used, with test duration of 2–21 days for fish and 1–7 days for arthropods, and EC50 values for biomass or growth for algae with 1–7 days of exposure time (Maltby et al. 2005).

**Table 4:** The active ingredients assessed by PRIMET, with acute and chronic ETRs, PEC, and PNEC values

Active ingredient	PEC_*n*_ water (μg/L)	PNEC_water-acute_ (μg/L)	ETR_*n* water-acute_	ETR_*n* water-chronic_
Alpha-cypermethrin	33.88–400.2	0.002821	12,000–142,000	623–7,366
Buprofezin	1.91–55.46	3.3	0.58–25.55	0.16–7.29
Butachlor	69.96–349.8	4.4	15.9–79.5	–
Diazinon	58.37–116.7	0.01	5,837–11,700	0.2–0.4
Ethoxysulfuron	35.27–86.69	19	1.85–4.56	0.01–0.02
Etofenprox	16.73–351.2	0.012	1,394–29,300	17.64–370.5
Fenclorim	4.19–93.26	6	0.70–15.54	–
Fenobucarb	5.8–652.4	1	5.8–652.4	–
Fenoxaprop-P-ethyl	111.2–270.9	1.9	58.55–142.6	0.01–0.09
Fipronil	92.37–325.6	1.9	48.62–171.4	11.26–39.69
Glyphosate	25.68	380	0.07	0.007
Hexaconazole	58.88–961.4	29	2.03–33.15	–
Imidacloprid	7.5–302.8	853	0.01–0.36	0.01–0.29
Iprodione	14.57–43.72	6.6	2.21–6.63	0.33–0.99
Isoprothiolane	77.69–333.2	68	1.14–4.9	–
Permethrin	97.48–146.2	0.006	16,300–24,400	5,090–7,635
Phenthoate	52.24	0.017	3,073	–
Propiconazole	8.69–181.2	9	0.96–20.13	0.27–5.72
Pyrazosulfuron ethyl	18.22–22.62	1,800	0.01	–
Quinalphos	36.04–108.1	0.0066	5,461–16,380	34.19–102.6
Quinclorac	87.68–109.7	298	0.29–0.36	–
Thiamethoxam	36.78–367.8	1,000	0.04–0.37	0.00–0.03
Tricyclazole	44.06	73	0.6	0.45

ETR values are classified as <1 = “no risk,” 1–100 = “possible risk,” and >100 = “definite risk” based on worst-case assumptions preset by PRIMET

EC_50_ and LC_50_ data were compiled from single-species aquatic toxicity tests including both static and flow-through tests and were log-transformed according to Raimondo et al. (2009). SSDs were computed with the software ETX 2.0 (Van Vlaardingen et al. 2003). The geometric means of EC_50_ and LC_50_ concentrations (μg/L) per species (if several tests) were inserted into the ETX software for each substance. The data points were then fitted to a log normal distribution model. The model fit was tested using the Anderson-Darling goodness-of-fit test with *α* = 0.05 as a critical level. A hazardous concentration (HC_5_ and HC_50_) affecting 5, respectively, 50% of the species under consideration was then obtained. The PEC_1_ from PRIMET (one single pesticide application) was used to estimate the exposure to the pesticides and provided a basis to calculate a PAF, based on the rice farmers’ pesticide use. Unlike the ETR quotients that are based on PEC_*n*_ values, PEC_1_ values were chosen for PAF calculations as they represent the environmental concentration of a single pesticide application, which was assessed to best correspond with the acute toxicity test that the SSDs are based on. The majority of the scenarios in PRIMET, however, only included one application. For the calculations of the PAFs, maximum and average concentrations per management regime and per province were used (Table 6).

### Comparison of risks between different management regimes and provinces

In order to get a more general comparison of the aquatic risk caused by the farming types and between the two provinces, annual ETR values were calculated for each type. Annual ETR values were calculated by adding up all individual ETR values from each pesticide application during all the crops in 1 year (two to three crops/year) in one rice field. For farmers with more than one rice field, an average for all rice fields was calculated, as the variation between rice fields of one farmer in comparison to variation between farmers was negligible. The total amount of applied pesticides per year was calculated the same way.

### Statistics

In order to test if there were statistical differences in the amount of pesticides used (kg A.I./ha) and in the risk to aquatic organisms (ETR values) between the two provinces and the management types, data was compared using ANOVA with a 2^3^ factorial design with *province*, *IPM*, and *fish* as fixed factors and active ingredient (g/ha) and ETR values as dependent variables. Data was log-transformed to create a better fit to a normal distribution, and the significance level for all tests was set to *α* = 0.05. Homogeneity of variance was tested with an *F* test. Non-parametric Kruskal-Wallis tests were used on data that had large variance (e.g., ETR comparison between provinces). Statistics were calculated using R (version 3.1.2).

## Results

### ETRs

ETR values could be calculated for 28 of the active ingredients, of which 13 were insecticides, 9 herbicides, and 6 fungicides. Five pesticides (bensulfuron-methyl, cartap, isoprocarb, pretilachlor, and validamycin) identified in the interviews could not be assessed with PRIMET, due to missing physico-chemical parameters in published literature and current databases, and were therefore not included in the analysis (Table 3). Pretilachlor was the most commonly used herbicide among the farmers (21 users), which often was found in a mixture with a herbicide safener, fenclorim.

Active ingredients that generated no or low risk to aquatic organisms (ETR <1) were the insecticides imidacloprid and thiamethoxam, the herbicides pyrasulfuron ethyl and quinclorac, and the fungicide tricyclazole. A possible risk (ETR 1–100) was found for the insecticide buprofezin; the herbicides butachlor, ethoxysulfuron, and fenclorim (herbicide safener); and the fungicides hexaconazole, iprodione, isoprothiolane, and propiconazole. Hexaconazole was the most commonly used fungicide among the farmers with 43 users and a total of 113 applications (15% of all applications) (Table 3).

High-risk pesticides, i.e., substances generating ETR values >100, were the insecticides, alpha-cypermethrin, diazinon, etofenprox, fenobucarb, fipronil, permethrin, phenthoate, quinalphos, and the herbicide fenoxaprop-P-ethyl, indicating a high risk for acute toxicity to aquatic organisms for these compounds at the predicted concentrations in rice paddies. The pyrethroids alpha-cypermethrin and permethrin also had high ETR values in the chronic assessment, while the chronic risk quotients for diazinon and fenoxaprop-P-ethyl were below 1. For fipronil, the chronic ETR values were lower than 100 but still constituted a possible risk. A chronic ETR value could not be calculated for fenobucarb and phenthoate due to missing input parameters (Table 4). Fenobucarb was the most commonly used insecticide in the study with a total of 55 users in both provinces. Out of the 154 application scenarios with fenobucarb (20% of all applications) (Table 3), 124 (80%) generated risk values above 100. For alpha-cypermethrin, diazinon, etofenprox, permethrin, phentoate, and quinalphos, all application scenarios generated acute ETR values above 100. For fipronil, 9 out of 17 application scenarios generated risk values above 100 and for the herbicide fenoxaprop-P-ethyl, 5 out of 8. In total, insecticides made up almost 50% of all application scenarios, of which 58% generated a risk value above 100.

### SSDs

Species sensitivity distributions were calculated for all substances, which generated ETR values above 100, except for the herbicide fenoxaprop-P-ethyl, which was excluded from further analysis, as there was too little algal toxicity data available for this substance.

The results indicate that the pyrethroids alpha-cypermethrin, etofenprox, and permethrin are the substances that are most likely to cause toxic effects on fish, with HC_5_/HC_50_ of 0.57/5.51, 0.91/282.95, and 0.95/8.19 μg/L, respectively. These were followed by phenthoate, fipronil, quinalphos, diazinon, and fenobucarb in descending order of toxicity (Table 5). For arthropods, the same insecticides were assessed, except for quinalphos, where too little toxicity data was available. Here, the pyrethroids permethrin and alpha-cypermethrin displayed the highest toxicity with HC_5_/HC_50_ of 0.0084/1.24 and 0.0085/3.11 μg/L, respectively, and fipronil 0.037/1.77 μg/L. The SSDs for fenobucarb and phentoate were only based on three data points each; hence, results should be cautiously interpreted, although phenthoate had the lowest HC_5_ value. Overall, all the insecticides were more toxic to arthropods than to fish.

**Table 5:** HC_5_ and HC_50_ concentrations to fish and arthropods calculated from species sensitivity distributions for the insecticides with ETR values >100

Active ingredient	Fish			Arthropod		
	No. of data points^a^	HC_5_ (μg/L)	HC_50_ (μg/L)	No. of data points^a^	HC_5_ (μg/L)	HC_50_ (μg/L)
Alpha-cypermethrin	6	0.57 (0.05–1.77)	5.51 (1.90–15.99)	5	0.0085 (0.00000274–0.21)	3.11 (0.31–73.52)
Diazinon	20^b^	505.4 (221–890.8)	3403 (2189–5291)	25	0.48 ( 0.18–0.95)	6.30 (3.70–10.74)
Etofenprox	7	0.91 (0.00–13.44)	282.95 (24.88–3218.48)	12	0.058 (0.0015–0.52)	21.01 (3.44–128.3)
Fenobucarb	7	570.6 (97.42–1349)	3577 (1642–7792)	3	1.14 (0.00028–7.47)	18.89 (1.64–217.9)
Fipronil	5	37.72 (4.83–85.22)	170.46 (75.97–382.50)	29	0.037 (0.0099–0.1)	1.77 (0.85–3.71)
Permethrin	16	0.95 (0.5–1.92)	8.19 (4.66–14.38)	54	0.0084 (0.0026–0.02)	1.24 (0.62–2.47)
Phenthoate	10	4.69 (0.48–17.12)	117.17 (39.16–350.55)	3	0.0056 (0.0000000000039–0.67)	7.07 (0.014–0.0035)
Quinalphos	11	120.3 (35.2–246.9)	774.8 (425.1–1412)	–	–	–

Values in brackets are the confidence intervals

^a^No of data points indicate the number of species used in each SSD

^b^Data did not pass the Anderson-Darling test on log-normality at the 5% level, but at 2.5%

Rice farmers, without fish, generally used more toxic compounds generating higher PAF values compared to rice-fish farmers (Table 6). For the less toxic substance fenobucarb and fipronil, these patterns were not as clear, although rice-fish farmers applying IPM had a somewhat lower PAF for fipronil compared to the other farmers. Fenobucarb had the lowest estimated fish toxicity and relatively low PAF compared to the other substances (Table 5 and 6). Alpha-cypermethrin had a PAF of more than 95% for all farmer groups, indicating that almost all fish species could be affected by the use of this insecticide. Thus, the use of alpha-cypermethrin probably poses a high risk to fish (PAN 2014; Lewis et al. 2016), but the results should be interpreted somewhat carefully as the SSD is only based on six data points (Table 5). Similarly, the SSD for fipronil was generated using five data points only. The calculated risks do not include application over seasons or the combined toxicity of multiple pesticides (i.e., mixture toxicity). Except for permethrin and alpha-cypermethrin (where more than 90% of the fish species were affected), the PAF of arthropods was noticeably higher than that of fish, indicating that a larger fraction of arthropod species compared to fish species were affected by the PECs of the study.

**Table 6:** The highest and average predicted environmental concentrations (PECs) per insecticide and management regime (provinces CT = Can Tho; TG = Tien Giang) and their associated potentially affected fraction (PAF) with confidence interval and standardized log concentration, calculated from the SSDs presented in Table 5

Active ingredient	Management	Maximum PEC1 (μg/L)	Fish		Arthropod	
			Median PAF (%)	Standard log concentration	Median PAF (%)	Standard log concentration
Alpha-cypermethrin	CT-R	400.2	99.90 (92.51–100)	3.31	91.26 (62.07–99.40)	1.46
	CT-RF	80.04	97.38 (78–99.94)	4	81.91 (50.65–96.80)	0.98
	CT-RIPM	160.1	99.26 (85.70–100)	4.88	86.52 (55.81–98.36)	1.19
	CT-RFIPM	53.36	95.03 (72.32–99.77)	3.48	79.24 (47.94–95.66)	0.87
Diazinon	CT-R	116.7	0.18 (0.01–1.92)	−2.96	96.79 (90.50–99.22)	1.87
	CT-RF	58.37	0.02 (0.00–0.58)	−3.56	92.10 (82.83–97.07)	1.43
Etofenprox	CT-R	351.2	52.49 (28.77–75.34)	0.07	78.43 (59.51–91)	0.81
	CT-RIPM	117.1	39.93 (18.68–64.75)	−2.67	68.47 (49.10–83.81)	0.49
	CT-RFIPM	16.73	20.74 (16–46.47)	−0.85	47.46 (29.49–65.97)	−0.07
Fenobucarb	CT-R	418.2	2.74 (0.11–19.67)	−2.03	96.46 (56.52–99.99)	2.14
	CT-RF	434.9	2.96 (0.09–20.29)	−1.99	96.64 (56.89–99.99)	2.16
	CT-RIPM	290	1.23 (0.06–14.49)	−2.37	94.51 (52.96–99.98)	1.88
	CT-RFIPM	241.6	0.8 (0–12.32)	−2.54	93.26 (51.09–99.96)	1.76
	TG-R	362.4	2.02 (0.12–17.5)	−2.16	95.78(55.16–99.99)	2.04
	TG-RF	652.4	6.35 (−0.09–27.59)	−1.61	98.03 (60.54–100)	2.44
	TG-RIPM	362.4	2.02 (0.12–17.5)	−2.16	95.78(55.16–99.99)	2.04
	TG-RFIPM	420.6	2.77 (0.11–19.76)	−2.02	96.49 (56.58–99.99)	2.14
Fipronil	CT-R	233.1	63.54 (33.85–86.69)	0.37	98.10 (93.86–99.57)	2.10
	CT-RF	233.1	63.54 (33.85–86.69)	0.37	98.10 (93.86–99.57)	2.10
	CT-RIPM	224.1	61.89 (32.48–85.57)	0.32	98.02 (93.69–99.54)	2.08
	CT-RFIPM	194.2	55.73 (27.53–81.20)	0.15	97.70 (93.04–99.44)	2.02
Permethrin	CT-R	146.2	98.6 (92.48–99.87)	2.24	94.17 (88.94–97.28)	1.58
Phenthoate	TG-R	52.24	33.90 (16.82–55.14)	−0.43	68.30 (29.19–93.60	0.54
Quinalphos	CT-R	108.1	4.1 (0.47–17.15)	−1.79	–	–
	CT-RF	36.04	0.34 (0.01–5.15)	−2.79	–	–
Active ingredient	Management	Average PEC1 (μg/L)	Fish		Arthropod	
			Median PAF (%)	Standard log concentration	Median PAF (%)	Standard log concentration
Alpha-cypermethrin	CT-R	285.86	99.79 (99.4–100)	3.05	89.69 (59.84–99.13)	1.36
	CT-RF	70.04	96.74 (76.23–99.91)	1.96	80.92 (49.62–96.39)	0.94
	CT-RIPM	100.19	98.22 (80.76–99.97)	2.24	84.10 (53.02–97.61)	1.07
	CT-RFIPM	48.02	94.20 (70.70–99.67)	1.67	78.14 (46.85–95.16)	0.83
Diazinon	CT-R	116.7	0.18 (0.01–1.92)	−2.96	96.79 (90.50–99.22)	1.87
	CT-RF	58.37	0.02 (0.00–0.58)	−3.56	92.10 (82.83–97.07)	1.43
Etofenprox	CT-R	292.7	50.39 (27.03–73.62)	0.01	76.92 (57.85–89.98)	0.75
	CT-RIPM	88.79	36.87 (16.38–62.04)	−0.35	65.77 (46.44–81.67)	0.42
	CT-RFIPM	16.73	20.74 (16–46.47)	−0.85	47.46 (29.49–65.97)	−0.07
Fenobucarb	CT-R	340.73	1.77 (0.11–16.63)	−2.22	95.46 (54.56–99.99)	1.99
	CT-RF	187.7	0.42 (0.00–9.75)	−2.78	92.52 (50.09–99.94)	1.69
	CT-RIPM	161.49	0.28 (0.00–8.43)	−2.92	89.72 (46.73–99.79)	1.48
	CT-RFIPM	186.4	0.41 (0.00–9.68)	−2.79	91.11 (48.32–99.88)	1.58
	TG-R	138.75	0.19 (0.00–7.24)	−3.07	88.39 (45.31–99.70)	1.39
	TG-RF	175.43	0.35 (0.00–9.14)	−2.85	91.39 (48.65–99.89)	1.60
	TG-RIPM	154.75	0.25 (0.00–8.08)	−2.96	89.28 (46.25–99.77)	1.45
	TG-RFIPM	223.4	0.66 (0.00–11.47)	−2.62	93.44 (51.34–99.96)	1.77
Fipronil	CT-R	230.1	63 (33.40–86.33)	0.35	98.07 (93.81–99.56)	2.09
	CT-RF	145.69	43.10 (17.96–71.55)	−0.19	96.95 (91.58–99.16)	1.89
	CT-RIPM	186.73	54.02 (26.19–79.93)	0.11	97.61 (92.86–99.40)	2.00
	CT-RFIPM	153.53	45.39 (19.63–73.35)	−0.12	97.10 (91.87–99.22)	1.92
Permethrin	CT-R	121.84	98.02 (90.86–99.77)	2.1	93.44 (87.91–96.83)	1.52
Phenthoate	TG-R	52.24	33.90 (16.82–55.14)	−0.43	68.30 (29.19–93.60	0.54
Quinalphos	CT-R	96.09	3.27 (0.32–15.31)	−1.9	–	–
	CT-RF	36.04	0.34 (0.01–5.15)	−2.79	–	–

### Comparison of risks between different farming strategies and provinces

**Fig. 3:**
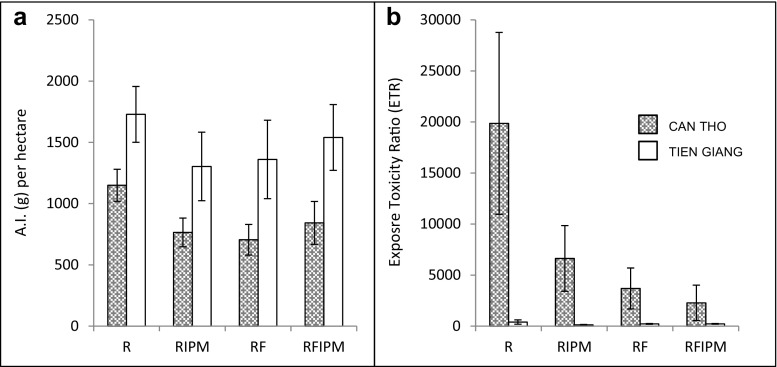
**a** Average applied active ingredients (g/ha) and **b** average acute ETR values calculated for the unit rice field for the two provinces Can Tho and Tien Giang. *Error bars* show the standard errors of the mean per province and management regime

Average amount of applied active ingredients (g/ha) and average acute ETR values in each province and for each farming type are presented in Fig. 3. Only pesticides that could be evaluated by PRIMET are included. Farmers in Tien Giang used significantly higher amount of active ingredients (1.5 times more) than farmers in Can Tho. There were, however, no significant differences between the log (ETR) data. Due to a significant difference in variance between provinces for log (ETR) values (*F* test, *α* = 10^−8^), the log-transformed ETR data was instead tested with a non-parametric Kruskal-Wallis test, in order to see if there were any effects of the management regimes within each province. The test did not reveal any significant differences between management regimes, *α* = 0.97 (Can Tho) and *α* = 0.16 (Tien Giang), and hence, no statistical differences for average ETR could be detected, although there was a trend indicating that rice-fish farmers generally showed a lower risk in their choice and use of pesticides as compared to rice farmers and that IPM farmers generally had a lower risk than non-IPM farmers in Can Tho, where the risks were more pronounced (Fig. 3b). Similar results were seen when comparing the maximum ETR values between different management regimes and areas.

## Discussion

An advantage of using the PRIMET model is that it requires fairly low input of data to make a first-tier risk estimate for different pesticides (Ansara-Ross et al. 2008; Malherbe et al. 2013). This kind of first-tier screening model was therefore seen as suitable method for this study, which dealt with a large set of data of farmers’ pesticide applications under different management regimes and aimed at comparing risks of different pesticide uses to aquatic organisms.

However, it should be noted that the methodology used by PRIMET underestimates the risks with neonicotinoids to freshwater arthropods due to *Daphnias*’ low sensitivity (PNEC 853 μg/L). Other arthropods such as the nymphs of mayflies have proven to be much more sensitive to imidacloprid (Roessink et al. 2013; Van den Brink et al. 2016). A study evaluating if acute first- and second-tier approaches recommended by the European Food Safety Authority (EFSA) have a sufficient degree of protection for different insecticide groups also concludes that neonicotinoids need to be further assessed as some of the approaches used were underprotective for a few substances (Van Wijngaarden et al. 2015). Therefore, the results of neonicotinoids for this study should be interpreted with caution.

The species sensitivity distribution model was chosen primarily to include risks to fish, as fish are generally underrepresented in other higher-tier models, such as the PERPEST model, based on microcosm and mesocosm studies (Van den Brink et al. 2002). SSD is, however, based on single-species tests, for one pesticide at the time, and does not take into account species interactions, indirect effects between species, and toxicity of pesticide mixtures. Still, risk assessment based on SSDs has been shown to be good first-tier risk assessment of acute risks of pesticides to aquatic organisms (e.g., Maltby et al. 2005; Van den Brink et al. 2006; Maltby et al. 2009).

The risk assessment (i.e., ETR values to aquatic organisms) revealed a different pattern than what was detected from only studying the amounts of applied active ingredients (Fig. 3). In Tien Giang, farmers applied on average 1.5 more active ingredients per hectare than in Can Tho. The risks, however, appear to be higher in Can Tho. This is because the farmers in Tien Giang used pesticides, which are less toxic to aquatic organisms, such as a carbamate (fenobucarb), while the farmers in Can Tho used several insecticides, which have a high acute toxicity such as pyrethroids (alpha-cypermethrin, permethrin, etofenprox) and the organophosphates diazinon and quinalphos. Thus, the difference in ETR values between Can Tho and Tien Giang observed here shows that it is not only the amount of A.I. that is important to consider when evaluating potential risks by different pesticides to aquatic organism but also to compare the intrinsic properties of the different pesticides such as their toxicity, persistence, and lipophilicity.

The ETR data also indicate that rice-fish farmers in Can Tho used lower amounts of high-risk pesticides than rice farmers. Also, farmers with IPM training chose less toxic pesticides, compared to non-IPM farmers, which has previously been shown among Asian farmers (FAO 2013). A previous study showed that rice-fish farmers tend to be more aware of the possible negative side effects from pesticides on fish and therefore select less toxic pesticides (Berg and Tam 2012). This is confirmed by the results of this study, where the PAFs of fish from pesticides were generally lower among fish farmers compared to farmers only growing rice. Fenobucarb was, however, an exception, most likely due to its relative low toxicity to fish. Fenobucarb is also one the most popular insecticides among the farmers, probably due to its efficiency against pests, price, and marketing (Berg and Tam 2012).

PECs can be assumed to be somewhat higher than measured concentrations in the field due to worst-case assumptions. It is also important to remember that they represent peak water concentrations after application, something that is seldom captured in monitoring studies. Still, the predicted average peak concentrations of this study (PEC is 139–341 μg/L for fenobucarb under different management regimes; Table 6) correspond well with measured fenobucarb concentrations in an experimental rice field 1 h after application (127 μg/L) (Tam et al. 2016a). The measured concentrations of fenobucarb were found after application using a dose commonly used by farmers in the area. Another study investigating field exposure of diazinon to fish after farmers’ normal practice in the Mekong Delta found diazinon concentrations of 130–170 μg/L 1 h after exposure (Cong et al. 2008). This is somewhat higher than the PECs of this study (PEC is 58.37–116.7 for diazinon under different management regimes; Table 6).

Overall, the potential affected fraction was quite high and indicates that fish may be at risk from the high pesticide use in the Mekong Delta. Aquatic organisms living in the rice fields are probably continuously exposed to elevated levels of many different pesticides during their entire life cycles because rice farming is applied throughout the year (Toan et al. 2013; Tam et al. 2016b). In addition, pesticides are easily spread to other areas through spray drift and irrigation channels, exposing organisms to a variety of different pesticides.

Although the estimated PAFs are based on worst-case scenarios, the toxicity data used to generate the SSDs for fish are based on LC_50_ studies, thus indicating severe impacts on the fish. It is likely that also, lower concentrations than those used in the worst-case scenarios would cause sub-lethal effects in fish. For example, Tam et al. (2015a) showed that that low levels of fenobucarb (30 μg/L) and chlorphyrifos (0.5 μg/L) caused a significant inhibition of brain AChE activity in climbing perch, causing both decreased growth and survival rates in the fish. Also, at lower concentrations, freshwater arthropods, which have been shown to have a higher sensitivity to the investigated insecticides, are affected, thereby indirectly affecting the fish, as these usually are complementary food for many fish species. The high PAFs of arthropods indicate that there also could be other serious effects on ecosystem functioning, such as decreased amount of natural predators to pest organisms. Microcosm and mesocosm studies of the investigated substances were rare, and only studies evaluating diazinon, etofenprox, and permethrin were found. For diazinon, PEC values of 58.37–116.7 μg/L (Table 4) can be compared to a calculated community NOEC of 4.3 μg/L (Giddings et al. 1996) and for etofenprox PEC values in the range of 16.73–351.2 μg/L (Table 4) to a community NOEC of 2 μg/L (Blake 2004). For permethrin, a reduction of diversity in zooplankton communities was noticed at 0.5 μg/L (Kaushik et al. 1985), which is much lower than the PECs of 97.48–146.2 μg/L found in this study.

The calculated risk from pesticide use found both from the SSD and the PRIMET models indicate high risks for aquatic organisms including fish, despite the fact that the models do not account for repeated application of pesticides (the fish are often stocked during two to three crops) or additive toxicity of substances applied together (toxicity from pesticide mixtures). Some of the pesticides identified from the interviews were also omitted from the analyses due to insufficient current knowledge on their toxic properties.

The aquaculture industry is currently expanding rapidly in the delta (De Silva and Phuong 2011) with increasing demands for a good water quality and healthy food products. The high use of pesticides in rice farming probably creates sub-optimal conditions for integrated rice-fish farming and may have negative implications for a sustained and healthy production of fish in the delta. The pesticide market in Vietnam has expanded during the last 10 years, and it is a challenge for the country’s large number of small-scale farmers to find appropriate technical advice from, e.g., retailers (Hoi et al. 2016), on a safe use of new pesticides. A group of pesticides that has steadily increased over the period of 1999–2013 (Hoi et al. 2016) falls under the classification II—moderately toxic (WHO 2010). Most of the substances with the highest toxicity of this study are included in that category. To reduce risks among farmers in the future, several policy options should be considered together, such as reducing pesticide supply, developing monitoring of pesticide risks in combination with lowering pesticide demand through, e.g., farmer field training (Schreinemacher et al. 2015).

Even though the use of PRIMET for probabilistic risk assessments is a good first step, there is a need to conduct further risk assessments in the area based on site-specific measured environmental concentrations (MECs) to better assess the risks for aquatic organisms in the field. Still, by applying an easy-to-use approach, pesticides with lower predicted risk can be identified and preferred in management. Furthermore, the methodology identifies some priority substances that would be good to monitor in the field to further evaluate their risks to the environment.

## Conclusions

Our study shows that farmers use a large number of pesticides likely to cause negative environmental effects on fish and other aquatic organisms. We therefore suggest that a screening model like PRIMET could provide a valuable tool to help provincial plant protection departments to select and recommend pesticides with potentially low environmental impacts and to substitute the pesticides that pose the highest risk with less toxic ones. Such screening models, with low data requirements, are especially relevant in developing countries, where field data are still scarce. Still, the lack of physico-chemical and toxicity data for some pesticides in this study was a problem and is something that needs to be further improved to make these kinds of first-order risk estimates operational in Vietnam and other developing countries. The combination of PRIMET with SSDs was a good way to assess fish toxicity based on PECs derived from interview data. Of highest concern for acute toxicity in this study were pyrethroids and a pyrazole pesticide and organophosphates. Farmers that had received IPM training and integrated rice-fish farmers often used pesticides with a lower risk compared to conventional rice farmers. Considering the increasing pesticide market and the large number of small-scale farmers in the country, governmental regulation of pesticides in combination with other strategies remain a key issue for reducing pesticide risks in the future.
